# HPAanalyze: an R package that facilitates the retrieval and analysis of the Human Protein Atlas data

**DOI:** 10.1186/s12859-019-3059-z

**Published:** 2019-09-09

**Authors:** Anh Nhat Tran, Alex M. Dussaq, Timothy Kennell, Christopher D. Willey, Anita B. Hjelmeland

**Affiliations:** 1 Department of Cell, Developmental and Integrative Biology, University of Alabama at Birmingham, THT 948, 1900 University Blvd, Birmingham, AL 35294 USA; 2 Department of Pathology, University of Alabama at Birmingham, 121 Shelby Biomedical Research Building, Birmingham, AL 35294 USA; 3 Department of Genetics, University of Alabama at Birmingham, 121 Shelby Biomedical Research Building, Birmingham, AL 35294 USA; 4 Department of Radiation Oncology, University of Alabama at Birmingham, 176 Facility Building, Birmingham, AL 35294 USA

**Keywords:** Human protein atlas, Proteomics, Visualization, Software

## Abstract

**Background:**

The Human Protein Atlas (HPA) aims to map human proteins via multiple technologies including imaging, proteomics and transcriptomics. Access of the HPA data is mainly via web-based interface allowing views of individual proteins, which may not be optimal for data analysis of a gene set, or automatic retrieval of original images.

**Results:**

HPAanalyze is an R package for retrieving and performing exploratory analysis of data from HPA. HPAanalyze provides functionality for importing data tables and xml files from HPA, exporting and visualizing data, as well as downloading all staining images of interest. The package is free, open source, and available via Bioconductor and GitHub. We provide examples of the use of HPAanalyze to investigate proteins altered in the deadly brain tumor glioblastoma. For example, we confirm Epidermal Growth Factor Receptor elevation and Phosphatase and Tensin Homolog loss and suggest the importance of the GTP Cyclohydrolase I/Tetrahydrobiopterin pathway. Additionally, we provide an interactive website for non-programmers to explore and visualize data without the use of R.

**Conclusions:**

HPAanalyze integrates into the R workflow with the *tidyverse* framework, and it can be used in combination with Bioconductor packages for easy analysis of HPA data.

**Electronic supplementary material:**

The online version of this article (10.1186/s12859-019-3059-z) contains supplementary material, which is available to authorized users.

## Background

The Human Protein Atlas (HPA) is a comprehensive resource for exploration of the human proteome which contains a vast amount of proteomics and transcriptomics data generated from antibody-based tissue micro-array profiling and RNA deep-sequencing [1–7]. The program has generated protein expression profiles in human non-malignant tissues, cancers, and cell lines with cell type-specific expression patterns via an innovative immunohistochemistry-based approach. These profiles are accompanied by a large collection of high-quality histological staining images that are annotated with clinical data and quantification. The database also includes classification of proteins into both functional classes (such as transcription factors or kinases) and project-related classes (such as candidate genes for cancer). Starting from version 4.0, the HPA includes subcellular localization profiles based on confocal images of immunofluorescent, stained cells. Together, these data provide a detailed picture of protein expression in human cells and tissues, facilitating tissue-based diagnostic and research.

Data from the HPA are freely available via proteinatlas.org, allowing scientists to access and incorporate the data into their research. Previously, the R package *hpar* has been created for fast and easy programmatic access of HPA data [8]. Here, we introduce *HPAanalyze*, an R package that aims to simplify exploratory data analysis from those data, as well as provide other functions complementary to *hpar*.

## Implementation

*HPAanalyze* is an R software package with GPL-3 license that is designed for easy retrieval and exploratory analysis of data from HPA. *HPAanalyze* allows users to quickly import data tables and xml files from HPA and provides a visual summary of the data. All staining images available in HPA can also be downloaded. Data can be obtained for single proteins or a protein set for pathway analysis. *jsHPAanalyze* is a JavaScript software suite with a GPL-3 license designed to create an interface in which non-programmers can simulate the R software package environment.

### The different HPA data formats

The HPA project provides data via two main mechanisms: Full datasets in the form of downloadable compressed Tab-Separated Value (TSV) files are available as well as individual entries in Extensible Markup Language (XML), Resource Description Framework (RDF), and TSV formats. The full downloadable datasets include normal tissue, pathology (cancer), subcellular location, RNA gene, and RNA isoform data. For individual entries, the XML format is the most comprehensive: it provides information on the target protein, antibodies, and a summary of each tissue. Also provided are detailed data from each sample including clinical information, immunohistochemistry (IHC) scoring, and image download links.

### HPAanalyze overview

**Fig. 1:**
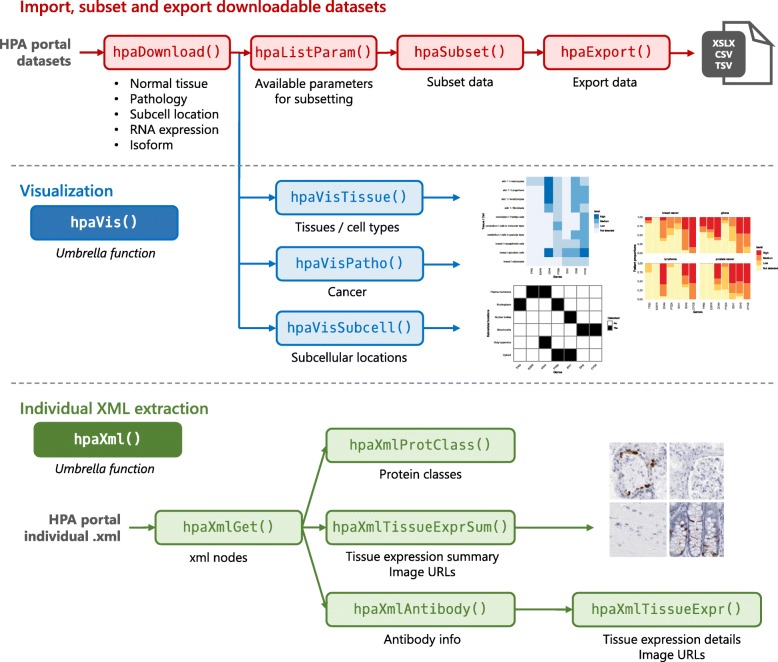
*HPAanalyze* Workflow. *HPAanalyze* provides functions for downloading, extracting and visualizing data from HPA. The functions are divided into three different families: (1) hpaDownload for downloadable datasets; (2) hpaVis for quick and customizable visualization; and (3) hpaXml for extracting information from individual XML files. Images shown are example data generated or images that can be downloaded from HPA (https://www.proteinatlas.org)

*HPAanalyze* is designed to fulfill three main tasks: (1) import, subsetting and export downloadable datasets; (2) visualization of downloadable datasets for exploratory analysis; and (3) facilitation of work with individual XML files (Fig. 1). This package aims to serve researchers with little programming experience, while also allowing power users to utilize the imported data as desired.

### Obtaining HPAanalyze

The stable version of *HPAanalyze* is available via Bioconductor and can be installed with the following code:

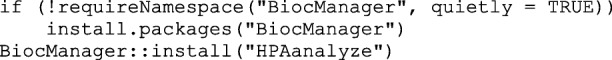



The development version of *HPAanalyze* is available on Github can be installed with the following code:

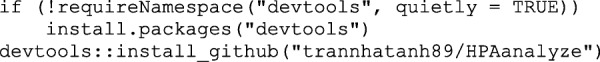



### Full dataset import, subsetting and export

The *hpaDownload* function downloads full datasets from HPA and imports them into R as a list of data frames (the *“tibble”*/ *tbl_df* variant commonly used in the *tidyverse* framework [9]). Data frames can subsequently be subset with *hpaSubset* and exported into XLSX, CSV or TSV formats with *hpaExport*. The standard object allows the imported data to be further processed in a traditional R workflow. The ability to quickly subset and export data gives researchers the option to use other non-R downstream tools, such as GraphPad for creating publication-quality graphics, or share a subset of data containing only proteins of interest.

### Visualization

With the intent to aid exploratory analysis, the *hpaVis* function family takes the output of *hpaDownload* (or *hpaSubset*) and provides quick visualization of the data. Nevertheless, the standard *ggplot* [10] object output of these functions gives users the option to further customize the plots for publication. All *hpaVis* functions share the same syntax for arguments: subsetting, specifying colors, and opting to use custom themes.

The first release of the *HPAanalyze* package includes three functions: *hpaVisTissue* for normal tissue samples, *hpaVisPatho* for the pathology/cancer samples, and *hpaVisSubcell* for subcellular localization data. All operations of this function family can be easily accessed through the umbrella function *hpaVis*.

### Individual XML import and image downloading

The *hpaXml* function family imports and extracts data from individual XML entries from HPA. The *hpaXmlGet* function downloads and imports data as an *“xml_document”/“xml_node”* object, which can subsequently be processed by other *hpaXml* functions. The XML format from HPA contains a wealth of information that may not be covered by this package. However, users can extract any data of interest from the imported XML file using the *xml2* package.

In the first release, *HPAanalyze* includes four functions for data extraction from HPA XML files: *hpaXmlProtClass* for protein class information, *hpaTissueExprSum* for summary of protein expression in tissue, *hpaXmlAntibody* for a list of antibodies used to stain for the protein of interest, and *hpaTissueExpr* for complete and detailed data from each sample including clinical data and IHC scoring. *hpaTissueExprSum* and *hpaTissueExpr* provide download links to obtain relevant staining images, with the former function also providing the option to automate the downloading process. Similar to the *hpaVis* family, all functionalities of this family may also be accessed through the simple umbrella function *hpaXml*.

### Compatibility with *hpar* Bioconductor package

*HPAanalyze* was designed to be compatible and complementary to other existing software packages. Table 1 shows the different capibilities of *HPAanalyze* and *hpar*, a Bioconductor package optimized for fast acquisition of subsets of HPA data.

**Table 1:** Complementary functionality between *hpar* and *HPAanalyze*

Functionality	*hpar*	*HPAanalyze*
Datasets	Included in package	Download from server or import from *hpar*
Query	Ensembl id	HGNC symbol and Ensembl id
Data version	One stable version	Latest by default, option to download older
Release info	Access via functions	Not applicable
View relevant browser page	Via getHPA function	Not applicable
Visualization	Not applicable	Exploratory via hpaVis functions
XML	Not applicable	Download and import via hpaXml functions
Histology image	View by loading browser page	Extract links via hpaXml functions

## Typical workflows and sample codes

The *HPAanalyze* package can be loaded with the following code:

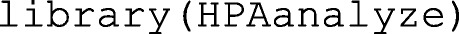



### Working with HPA downloadable datasets

Using *HPAanalyze*, a typical workflow with HPA downloadable datasets consists of the following steps:

1. Download and import data into R with *hpaDownload*.

2. View available parameters for subsetting with *hpaListParam*.

3. Subset data with *hpaSubset*.

4. Optional: Export data with *hpaExport* (Fig. 1).

The following code can be used to download the histology datasets (normal tissue, pathology, and subcellular location).

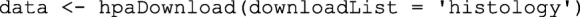



The output of the code shows that data can be subset by normal tissue types, normal cell types, cancer types, and subcellular location. The “normal_tissue” dataset contains information about protein expression profiles in human tissues based on IHC staining. The “pathology” dataset contains information about protein expression profiles in human tumor tissue based on IHC staining with the number of patients annotated for four staining levels together with log-rank *p* values for survival/mRNA correlation. The “subcellular_location” dataset contains information about subcellular localization of proteins based on immunofluorescence (IF) staining of normal cells.

*hpaListParam* function prints a list of available parameters that can be used to subset the downloaded datasets. Below are the first three items in each group:

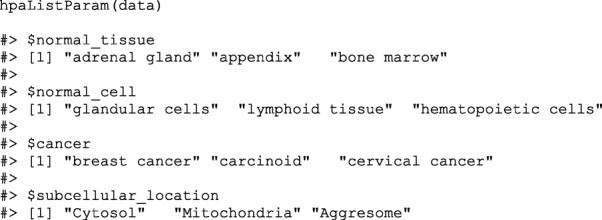



Based on the information, the downloaded data may be subset based on genes, tissues, cells and subcellular locations of interest. As an example, the following code filters the datasets for MKI67 (Ki67), breast tissue, and breast cancer.

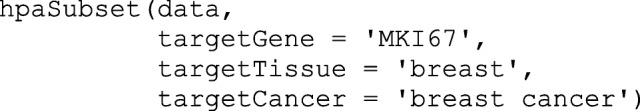



The results (below) showed that Ki67 is expressed at non-detectable-to-medium levels in normal breast tissue, but medium-to-high levels in breast cancer. The data also indicated, with high reliability, that Ki67 is expressed at high levels in the nuclear bodies, nucleoli and nucleus.

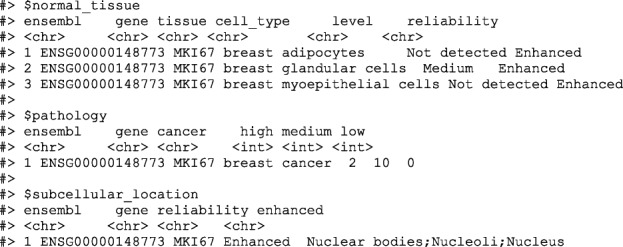



We next sought to facilitate the downstream analysis of data using a non-R software as well as the storage of data subsets for reproducible research. To accomplish this goal, the *HPAanalyze* package included the *hpaExport* function. The *hpaExport* function exports data into Excel file format, with each sheet for a dataset. As an example, the code to export the above Ki67 data and generate an .xlsx file called ‘ki67.xlsx’ is as noted below.

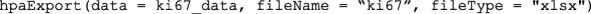



### Visualization with the hpaVis function family

With the goal of aiding exploratory analysis of a group of target proteins, *HPAanalyze* provides the ability to quickly visualize data from downloaded HPA datasets with the *hpaVis* function family (Fig. 1). These functions maybe particularly useful for gaining insights into pathways or gene signatures of interest.

The *hpaVis* functions share a common syntax, where the input is the object generated by *hpaDownload* or *hpaSubset*. Depending on the function, the target arguments allows the user to choose to visualize vectors of genes, tissue, cell types, etc. All *hpaVis* functions generate standard *ggplot2* plots, which allow further customization of colors and themes. Currently, the normal tissue, pathology, and subcellular localization data can be visualized.

**Fig. 2:**
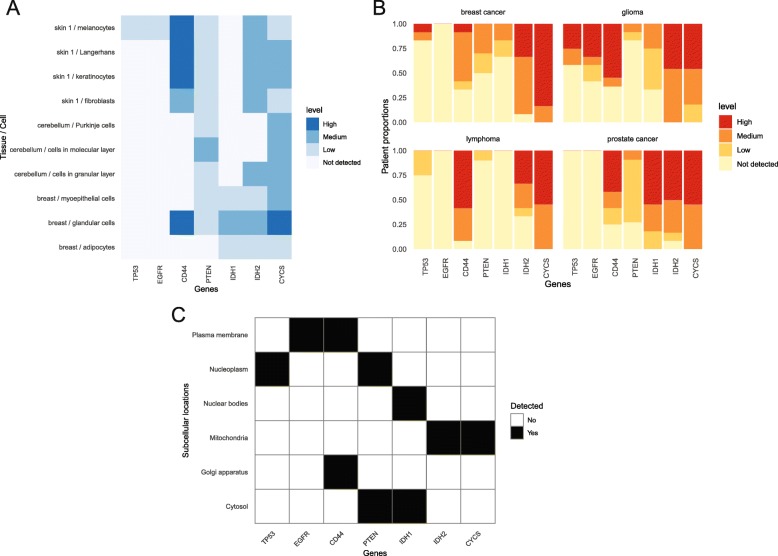
Example of *hpaVis* Function Family. **a** *hpaVisTissue* generates a heatmap of protein expression levels in tissues and cells of interest. **b** *hpaVisPatho* creates multiple bar graphs of protein expression in individual target cancers. **c** *hpaVisSubcell* visualizes subcellular localization of proteins of interest in a tile chart. Proteins of interest shown via gene name include those implicated in cancer of known subcellular localization: patterns of expression were visualized for Tumor Protein p53 (TP53), Epidermal Growth Factor Receptor (EGFR), Cluster of Differentiation 44 (CD44), Phosphatase and Tensin Homolog (PTEN), Isocitrate Dehydrogenase 1 (IDH1), Isocitrate Dehydrogenase 2 (IDH2), and Cytochrome C (CYCS)

*hpaVisTissue* generates a “heatmap”, in which the expression of proteins of interest as measured with quantified IHC staining is plotted for each cell type of each tissue (Fig. 2a). *hpaVisPatho* generates an array of column graphs showing the expression of proteins of interest in each cancer (Fig. 2b). *hpaVisSubcell* generates a tile chart showing the subcellular localization of proteins of interest (Fig. 2c).

### Working with individual XML files for each target protein

The *hpaXml* function family supports importing and extracting data from individual XML files provided by HPA for each protein. A typical workflow for use of XML files includes the following steps:

1. Download and import XML file with *hpaXmlGet*.
2. Extract the desired information with other *hpaXml* functions.
3. Download images of histological stains as currently supported by the *hpaXmlTissurExpr* and *hpaXmlTissueExprSum* functions (Fig. 1).

The *hpaXmlGet* function takes one HGNC symbol or Ensembl id (starting with ENSG) and imports the perspective XML file into R. This function calls the *xml2::read_xml* function under the hood, hence the resulting object may be processed further with functions from the *xml2* package if desired. The protein class of a queried protein can be extracted from the imported XML with *hpaXmlProtClass*. The function *hpaXmlTissueExprSum* extracts the summary of expression of a protein of interest in normal tissue. The output of this function is (1) a string containing a one-sentence summary, and (2) a data frame of all tissues in which the protein was positively stained and images of those tissues.

The XML files are the only format of HPA programmatically accessible data that contains information about each antibody and each tissue sample used in the project. *hpaXmlAntibody* extracts the antibody information and returns a data frame with one row for each antibody. *hpaXmlTissueExpr* extracts information about all samples for each antibody above and returns a list of data frames. If an antibody has not been used for IHC staining, the returned data frame will be empty. Each data frame contains clinical data (patientid, age, sex), tissue information (snomedCode, tissueDescription), staining results (staining, intensity, location) and one imageUrl for each sample.

### *jsHPAnalyze*: availability and use

We implemented the *hpaVis* function family in JavaScript, and this is available on GitHub at: https://github.com/adussaq/jsHPAanalyze. A tutorial on how to utilize the tool is available on YouTube at: https://youtu.be/9mZj7NJKiAE. We utilized bootstrap 4.3.1 for the page layout, apex charts 3.6.12 for figure creation, and Dexie 2.0.4 for data caching. To utilize the tool a user needs to limit the list of genes to less than 50. This can be done by selecting sections from each of the charts and by manually selecting genes by name. Once the user has limited the list, all three figures can be produced from the *hpaVis* function family.

## Results

To demonstrate the potential uses of *HPAanalyze*, we performed example case studies. Each case study was chosen based on the availability of a body of literature that could be used to validate functionality by demonstrating how the resulting data might confirm or complement cancer research.

### Case study 1: Glioma pathway alteration at the protein level

**Fig. 3:**
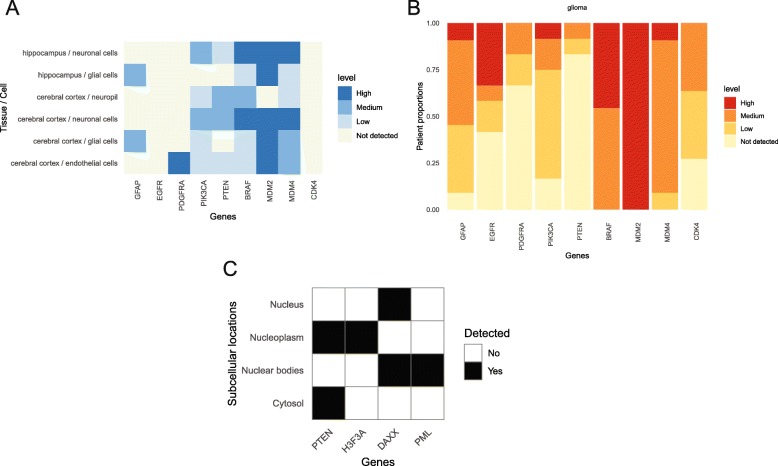
Case Study for Proteins of Interest in Glioblastoma. Expression of proteins frequently altered in glioblastoma in normal (**a**) or tumor (**b**) brain tissue. **c** Subcellular localization of proteins associated with non-canonical PTEN function. Molecules of interest shown include Glial Fibrillary Acidic Protein (GFAP), Epidermal Growth Factor Receptor (EGFR), Platelet-derived Growth Factor Receptor Alpha (PDGFRA), Phosphatidylinositol-4,5-Bisphosphate 3-Kinase Catalytic Subunit Alpha (PIK3CA), Phosphatase and Tensin Homolog (PTEN), v-Raf Murine Sarcoma Viral Oncogene Homolog B (BRAF), Mouse Double Minute 2-Like p53 Binding Protein (MDM2), Mouse Double Minute 4 Homolog (MDM4), Cyclin Dependent Kinase 4 (CDK4), H3 Histone Family Member 3A (H3F3A), Death Domain Associated Protein (DAXX), and Promyelocytic Leukemia Protein (PML)

As further detailed below, previous studies elucidating pathway alterations in glioblastoma (GBM) have identified frequent amplifications, deletions and mutations of certain genes belonging to Phosphatidylinositol-3-kinase (PI3K)/Mitogen-activated Protein Kinase (MAPK), p53 and Retinoblastoma Protein (Rb) pathways. The level of these proteins in normal brain (hippocampus and cerebral cortex) (Fig. 3a) and glioma (Fig. 3b) was therefore visualized with *HPAanalyze*. These data were acquired by quantification of IHC stained specimens, so levels of mutant proteins may not be represented. Furthermore, the cancer dataset contains data for all glioma: information about each specific cancer grade is only available via further examination of individual antibodies, which is outside of the scope of this case study.

As a positive control, we first evaluated the expression of Glial Fibrillary Acidic Protein (GFAP), a marker for astrocytes/glial cells. In the normal dataset, GFAP expression was found only on glial cells, suggesting that a false positive is unlikely (Fig. 3a). In glioma datasets, GFAP was found to be expressed at mostly medium to high levels (Fig. 3b), which is also consistent with the literature.

According to the TCGA data for GBM patients, approximately 90% of patients have alterations in the PI3K/MAPK pathway: Epidermal Growth Factor Receptor (EGFR), Platelet-derived Growth Factor Receptor Alpha (PDGFRA), and PI3K genes are frequently amplified or mutated to gain function (approximately 57, 10 and 25%, respectively), while Phosphatase and Tensin Homolog (PTEN) is deleted or mutated in 41% of patients [11]. Data from *HPAanalyze* supports this pattern, although the proportions are not identical (Fig. 3a-b). Differences can be attributed to the distinctions between target molecules (DNA/mRNA in TCGA versus protein in HPA) and the number of specimens. One example of the difference can be observed with data regarding v-Raf Murine Sarcoma Viral Oncogene Homolog B (BRAF). BRAF is only amplified/mutated in 2% of TCGA patients, but it is expressed at medium to high levels in all glioma specimens in HPA (Fig. 3b).

The p53 pathway is altered in 86% of GBM patients, with amplification of MDM2 (7.6%) and MDM4 (7.2%) leading to the inhibition of p53, which is also highly mutated [11]. The amplification of MDM2 and MDM4 is reflected at protein levels in HPA: MDM2 is expressed at high levels in all patients and MDM4 at medium levels in most patients (Fig. 3b). Similarly, the Rb pathway inhibitor CDK4 was found to be amplified in 14% of patient samples and confirmed by the stark contrast between normal and cancerous samples in HPA (Fig. 3a-b). The protein CDK4 is not detected in any normal brain cell, while it is present at some level in most glioma samples. These data confirm that *HPAanalyze* may be useful for comparison of normal and tumor tissue in order to identify or validate molecules of interest with altered expression in cancer.

### Case study 2: PTEN’s novel function through chromatin-associated complexes

PTEN is known as a key tumor suppressor which is frequently mutated in GBM [11]. Canonically, the protein functions as a phosphatase to dephosphorylate phosphatidylinositol (3,4,5)-trisphosphate (PIP_3_), which leads to inhibition of Akt signaling [12]. Akt is central to many hallmarks of cancer by promoting cell survival via inhibition of the apoptotic protein Bad, overcoming cell cycle arrest, facilitating glucose metabolism, inhibiting autophagy via regulation of the lysosomal biogenesis controller TFEB, and promoting tumor angiogenesis [13].

Since PIP_3_ is a phospholipid that resides on the plasma membrane [13], PTEN was once thought to act solely in the cytoplasm. However, a recently published study demonstrated that PTEN also forms complexes with the histone chaperone DAXX and the histone variant H3.3, modulating chromatin association to regulate oncogene expression. This effect is independent of PTEN enzymatic activity [14]. Congruent with these data, we noted that PTEN was present in both the cytosol and the nucleus (Fig. 3c) in HPA data, suggesting a non-canonical function for PTEN. The subcellular localization of DAXX and H3.3, as well as PML (which interacts with DAXX and regulates PTEN), further corroborate the newly discovered model of PTEN-DAXX-H3.3 gene regulation (Fig. 3c).

HPA subcellular localization information for individual proteins is acquired via immunofluorescent staining of human cell lines [3]. Therefore, the data do not account for various physiological conditions that may relocate proteins nor do the data directly provide evidence of protein-protein interactions. A query of HPA should always be followed by a confirmation study to ensure the validity of the results in any cell type or cancer of interest. Nevertheless, *HPAanalyze* offers a powerful approach to quickly explore curated and validated antibody-based protein expression data.

### Case study 3: Protein expression of GTP Cyclohydrolase I (GCH1)/tetrahydrobiopterin (BH4) pathway members

Summarized datasets regarding the expression of one protein may not be sufficient to understand the potential role of a pathway in normal or cancer tissue. We recently defined a role for GTP Cyclohydrolase I (GCH1), the first and rate limiting enzyme in the tetrahydrobiopterin (BH4) pathway, as an important regulator of glioblastoma growth [15]. The GCH1/BH4 pathway can regulate the production of reactive species, which can be pro- or anti-tumorigenic depending on a number of factors which we and others have reviewed [16]. In addition to GCH1 and the final product BH4, the de novo pathway also involves 6-pyruvoyltetrahydropterin synthase (PTS) and sepiapterin reductase (SPR), the latter of which has been known to be targeted by multiple well-established sulfa drugs [17]. BH4 can also be produced via the salvage pathway in which the oxidized product BH2 is converted back to BH4 by dihydrofolate reductase (DHFR) [18].

**Fig. 4:**
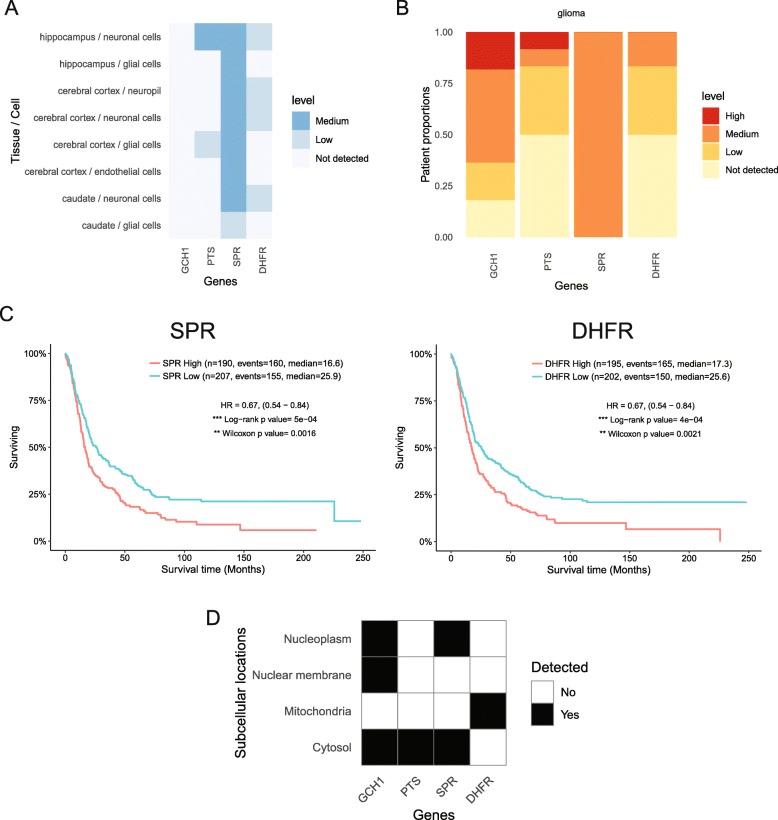
GTP Cyclohydrolase I/BH4 Pathway as a Potential Target for Glioma Research. **a**-**b** Protein expression data of genes in the GCH1/BH4 pathway from *HPAanalyze* in **a** normal brain tissue and **b** glioma. **c** Kaplan-Meyer survival curves showing a correlation of higher SPR and DHFR expression with worse glioma patient survival, data from the REMBRANDT dataset. Plots were generated using the GlioVis portal (http://gliovis.bioinfo.cnio.es) [19] **d** Subcellular location data for GCH1/BH4 pathway members from *HPAanalyze*

Using *HPAanalyze*, we confirmed our published finding that GCH1 was elevated in glioma in comparison to non-tumor brain tissue (Fig. 4a-b, Additional files 1 and 2). *HPAanalyze* indicated that GCH1 was not expressed in normal brain (cerebellum, cortex, hippocampus or caudate) (Fig. 4a). However, GCH1 was detected in glioma samples, and increased with tumor grades (Fig. 4b). Our analysis using the HPA datasets showed that, while SPR expression was relatively consistent between normal brain and tumor tissues, the other three members of the pathway were expressed at higher levels in glioma tumors than in normal cells (Fig. 4a-b). Survival analysis also demonstrated that, similar to our published findings with GCH1, elevated expression of SPR or DHFR correlated with worse survival of glioma patients (Fig. 4c, Additional files 3 and 4). Together, these data strengthen the notion that the BH4 pathway is important in glioma growth and suggest additional targets for therapeutic intervention, including those with readily available small molecule inhibitors.

The *hpaVisSubcell* function of *HPAanalayze* revealed an interesting aspect of the GCH1/BH4 pathway protein expression that is worthy of additional investigation. All members of the de novo biosynthesis pathway were expressed in the cytosol where they are expected to function as enzymes to produce BH4. However, GCH1 and SPR were also present in the nucleus (Fig. 4d), which may suggest additional roles as in transcriptional regulation.

### Case study 4: Glucose transporter 3 (GLUT3/ SLC2A3) in normal brain and glioma

To explore the capability of HPAanalyze to retrieve details of proteins of interest from HPA, we focused on GLUT3 (encoded by the gene SLC2A3) which facilitates the transport of glucose through cell plasma membranes. Together with other proteins in its family, GLUT3 plays an important role in regulating the metabolism in mammalian cells. In many cancers, including glioma, metabolic abnormality has been shown to promote tumor growth and maintenance [20]. In fact, GLUT3 inhibitors have been investigated as potential therapy for glioma [21]. Using the *hpaSubset* function, we found that GLUT3 expression was not detected in glial cells in the brain, while about a third of the glioma patients in the HPA datasets had GLUT3 expression in their tumors (Additional files 1 and 2).

**Fig. 5:**
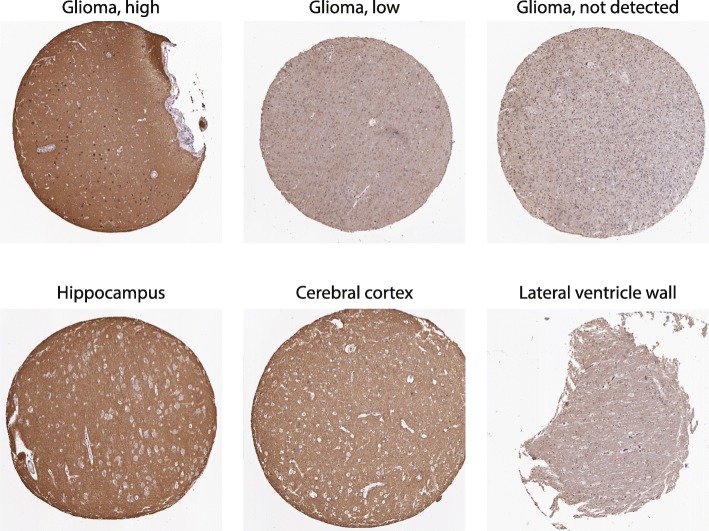
Expression of GLUT3 in the brain. Representative images of GLUT3 staining in normal brain and glioma tissues. The images are downloaded as detailed in the Additional files 1 and 2 using *HPAanalyze* and additional R codes

To acquire more details, we used the *hpaXmlGet* function to download the corresponding XML file for GLUT3. The *hpaXmlAntibody* function revealed that the HPA program had used two different antibodies to stain for GLUT3. Using the *hpaXmlTissueExpr* function, we were able to extract the full record of every staining available for both antibodies, including clinical data, staining quantifications, and links to the original images (Additional files 1 and 2). From there, we were able to filter the records and download GLUT3 staining images for normal brain tissue and glioma samples (Fig. 5). Those high-quality images may be further assessed by pathologists for information not available from HPA.

## Conclusions

We report the development of the R package *HPAanalyze*, which we believe will be highly useful for investigators interested in visualizing the expression data from HPA for signaling pathways. Using our R package *HPAanalyze*, we are able to retrieve, visualize and export data from the HPA program. Additionally, we created jsHPAanalyze which allows for non-programmers, with nothing more than a modern browser, to be able to create the visualizations described in this publication. We have new functionality compared to other available packages in that we can visualize the data as well as quickly download histological images of interest. Although it is a programmatic approach, which requires basic R programming skills, *HPAanalyze* was built with ease of use and reproducibility in mind, which makes the workflow and syntax very simple and straight-forward. With the case studies, we have also demonstrated how *HPAanalyze* can be easily integrated into different areas of research to identify new targets or provide more evidence for a working hypothesis. This software package is highly supportive of our research, and we plan to update it with new features and ensure future compatibility with the HPA program.

## Availability and requirements

### HPAanalyze

- Project name: HPAanalyze
- Project home page: https://github.com/trannhatanh89/HPAanalyze
- Operating system(s): All platforms where R is available, including Windows, Linux, OS X
- Programming language: R
- Other requirements: R 3.5.0 or higher, and the R packages dplyr, openxlsx, ggplot2, readr, tibble, xml2, tidyr, stats, utils, hpar, gridExtra
- License: GPL-3
- Any restrictions to use by non-academics: Freely available to everyone

### jsHPAanalyze

- Project name: jsHPAanalyze
- Project home pages: https://github.com/adussaq/jsHPAanalyze
- Operating system(s): All platforms where a modern browser is available, including Windows, Linux, OS X
- Programming language: JavaScript
- Other requirements: Modern browser such as Chrome or Firefox
- License: GPL-3
- Any restrictions to use by non-academics: Freely available to everyone

## Additional files

Additional file 1: R Codes for Figs. 2, 3, 4 and 5 html. Supplemental material for Figs. 2, 3, 4 and 5 html format. Input parameters for HPAanalyze to generate Figs. 2, 3, 4 and 5 are shown. (HTML 626 kb)

Additional file 2: R Codes for Figs. 2, 3, 4 and 5 rmd. Supplemental material for Figs. 2, 3, 4 and 5 rmd format. Input parameters for HPAanalyze to generate Figs. 2, 3, 4 and 5 are provided. (RMD 8 kb)

Additional file 3: SPR. Supplemental material for Fig. 4. Primary data in GlioVis used to generate the survival curves demonstrating that elevated SPR mRNA expression correlates with poor glioma patient survival. (CSV 26 kb)

Additional file 4: DHFR. Supplemental material for Fig. 4. Primary data in GlioVis used to generate the survival curves demonstrating that elevated DHFR mRNA expression correlates with poor glioma patient survival. (CSV 26 kb)

## Data Availability

All data analyzed during this study are publicly available at https://www.proteinatlas.org. The R package is available at https://github.com/trannhatanh89/HPAanalyze.

## Abbreviations

BRAF: v-Raf Murine Sarcoma Viral Oncogene Homolog B
CD44: Cluster of Differentiation 44
CDK4: Cyclin Dependent Kinase 4
CSV: Comma-Separated Values
CYCS: Cytochrome C
DAXX: Death Domain Associated Protein
DHFR: Dihydrofolate Reductase
EGFR: Epidermal Growth Factor Receptor
GBM: Glioblastoma
GCH1: GTP Cyclohydrolase I
GFAP: Glial Fibrillary Acidic Protein
GLUT3/SLC2A3: Glucose Transporter 3; Solute Carrier Family 2 Member 3
H3F3A: H3 Histone Family Member 3A
HGNC: HUGO Gene Nomenclature Committee
HPA: Human Protein Atlas
IDH1: Isocitrate Dehydrogenase 1
IDH2: Isocitrate Dehydrogenase 2
IF: Immunofluorescence
IHC: Immunohistochemistry
MAPK: Mitogen-activated Protein Kinase
MDM2: Mouse Double Minute 2-Like p53 Binding
MDM4: Mouse Double Minute 4 Homolog
MTX: Methotrexate
PDGFRA: Platelet-derived Growth Factor Receptor Alpha
PI3K: Phosphatidylinositol-3-kinase
PIK3CA: Phosphatidylinositol-4,5-Bisphosphate 3-Kinase Catalytic Subunit Alpha
PML: Promyelocytic Leukemia Protein
PTEN: Phosphatase and Tensin Homolog
PTS: 6-pyruvoyltetrahydropterin synthase
Rb: Retinoblastoma Protein
RDF: Resource Description Framework
SPR: Sepiapterin Reductase
TCGA: The Cancer Genome Atlas
TP53: Tumor Protein p53
TSV: Tab-Separated Value
XML: Extensible Markup Language
