# Supplementary material for: HPAanalyze: an R package that facilitates the retrieval and analysis of the Human Protein Atlas data
Source: BMC Bioinformatics. 2019 Sep 9;20:463. doi: 10.1186/s12859-019-3059-z (PMC6734269; doi:10.1186/s12859-019-3059-z)
Supplement: Supplementary file 1 — R Codes for Figs. 2, 3, 4 and 5 html. Supplemental material for Figs. 2, 3, 4 and 5 html format. Input parameters for HPAanalyze to generate Figs. 2, 3, 4 and 5 are shown. (HTML 626 kb) [file 12859_2019_3059_MOESM1_ESM.html]

Supplemental material


# Supplemental material

```
library(BiocStyle)
library(HPAanalyze)
library(dplyr)
library(ggplot2)
```

```
data <- hpaDownload(downloadList = "histology", 
                    version = "v18")
```

# Figure 2

```
gene_list_2 <- c("TP53", "EGFR", "CD44", "PTEN", "IDH1", "IDH2", "CYCS")

# Panel 2A
tissue_list_2 <- c("skin 1", "cerebellum", "breast")

plot_2a <-
  hpaVisTissue(data = data,
               targetGene = gene_list_2,
               targetTissue = tissue_list_2,
               color = c("#eff3ff", "#bdd7e7","#6baed6", "#2171b5"))

ggsave(filename = "plot_2a.pdf",
       plot = plot_2a,
       device = "pdf")

# Panel 2B
cancer_list_2 <- c("breast cancer", "glioma", "lymphoma", "prostate cancer")

plot_2b <-
  hpaVisPatho(data = data,
              targetGene = gene_list_2,
              targetCancer = cancer_list_2)

ggsave(filename = "plot_2b.pdf",
       plot = plot_2b,
       device = "pdf",
       width = 7,
       height = 5)

# Panel 2C
plot_2c <-
  hpaVisSubcell(data = data,
                targetGene = gene_list_2,
                color = c("white", "black"),
                reliability = c("enhanced", "supported", "approved"))

ggsave(filename = "plot_2c.pdf",
       plot = plot_2c,
       device = "pdf")
```

# Figure 3

```
gene_list_3 <- 
  c("GFAP", "EGFR", "PDGFRA", "PIK3CA", "PTEN", "BRAF", "MDM2", "MDM4", "CDK4")

# Panel 3A
tissue_list_3 <- c("hippocampus", "cerebral cortex")

plot_3a <-
  hpaVisTissue(data = data,
               targetGene = gene_list_3,
               targetTissue = tissue_list_3,
               color = c("#eff3ff", "#bdd7e7","#6baed6", "#2171b5"))

ggsave(filename = "plot_3a.pdf",
       plot = plot_3a,
       device = "pdf",
       width = 7,
       height = 5)

# Panel 3B
plot_3b <-
  hpaVisPatho(data = data,
              targetGene = gene_list_3,
              targetCancer = "glioma")

ggsave(filename = "plot_3b.pdf",
       plot = plot_3b,
       device = "pdf",
       width = 7,
       height = 5)

# Panel 3C
gene_list_3c <- c("PTEN", "H3F3A", "DAXX", "PML")

plot_3c <-
  hpaVisSubcell(data = data,
                targetGene = gene_list_3c,
                color = c("white", "black"),
                reliability = c("enhanced", "supported", "approved"))

ggsave(filename = "plot_3c.pdf",
       plot = plot_3c,
       device = "pdf",
       width = 4,
       height = 3)
```

# Figure 4

```
gene_list_4 <- c("GCH1", "PTS", "SPR", "DHFR")

# Panel 4A
tissue_list_4 <- c("hippocampus", "cerebral cortex", "caudate")

plot_4a <-
  hpaVisTissue(data = data,
               targetGene = gene_list_4,
               targetTissue = tissue_list_4,
               color = c("#eff3ff", "#bdd7e7","#6baed6", "#2171b5"))

ggsave(filename = "plot_4a.pdf",
       plot = plot_4a,
       device = "pdf",
       width = 5,
       height = 4)

# Panel 4B
plot_4b <-
  hpaVisPatho(data = data,
              targetGene = gene_list_4,
              targetCancer = "glioma")

ggsave(filename = "plot_4b.pdf",
       plot = plot_4b,
       device = "pdf",
       width = 5,
       height = 4)

# Panel 4C
# Figure was generated with the GlioVis portal http://gliovis.bioinfo.cnio.es/
# Accessed: June 19, 2019
#
# Plotting:
# Navigate through tabs: Explore > Survival > Kaplan-Meier > Plot
#
# Parameters: 
#    - Dataset: Adult Rembrandt
#    - Gene: SPR or DHFR
#    - Histology: All
#    - Subtype: All
#    - Cutoff: Median
#    - Plot options: use default options
#    - Download: use default options
#
# Retrieving plotting data: (same parameters)
# Navigate through tabs: Explore > Survival > Kaplan-Meier > Plot
# Buttons: Download > CSV

# Panel 4D
plot_4d <-
  hpaVisSubcell(data = data,
                targetGene = gene_list_4,
                color = c("white", "black"),
                reliability = c("enhanced", "supported", "approved"))

ggsave(filename = "plot_4d.pdf",
       plot = plot_4d,
       device = "pdf",
       width = 4,
       height = 3)
```

# Figure 5

```
hpaSubset(data = data,
          targetGene = "SLC2A3",
          targetTissue = c("hippocampus", "cerebral cortex", "caudate"),
          targetCellType = "glial cells",
          targetCancer = "glioma")

# $normal_tissue
# # A tibble: 3 x 6
#   ensembl         gene   tissue          cell_type   level        reliability
#   <chr>           <chr>  <chr>           <chr>       <chr>        <chr>      
# 1 ENSG00000059804 SLC2A3 caudate         glial cells Not detected Approved   
# 2 ENSG00000059804 SLC2A3 cerebral cortex glial cells Not detected Approved   
# 3 ENSG00000059804 SLC2A3 hippocampus     glial cells Not detected Approved   
# 
# $pathology
# # A tibble: 1 x 11
#   ensembl gene  cancer  high medium   low not_detected prognostic_favo~
#   <chr>   <chr> <chr>  <dbl>  <dbl> <dbl>        <dbl>            <dbl>
# 1 ENSG00~ SLC2~ glioma     1      2     1            8               NA
# # ... with 3 more variables: unprognostic_favorable <dbl>,
# #   prognostic_unfavorable <dbl>, unprognostic_unfavorable <dbl>
# 
# $subcellular_location
# # A tibble: 1 x 11
#   ensembl gene  reliability enhanced supported approved uncertain single_cell_var~
#   <chr>   <chr> <chr>       <chr>    <chr>     <chr>    <chr>     <chr>           
# 1 ENSG00~ SLC2~ Approved    NA       NA        Plasma ~ NA        NA              
# # ... with 3 more variables: single_cell_var_spatial <chr>,
# #   cell_cycle_dependency <chr>, go_id <chr>


SLC2A3xml <- hpaXmlGet("SLC2A3", version = "v18")

SLC2A3_ab <- hpaXmlAntibody(SLC2A3xml)
SLC2A3_ab
#   id        releaseDate releaseVersion RRID      
#   <chr>     <chr>       <chr>          <chr>     
# 1 CAB002763 2006-03-13  1.2            NA        
# 2 HPA006539 2008-02-15  3.1            AB_1078984

SLC2A3_expr <- hpaXmlTissueExpr(SLC2A3xml)
str(SLC2A3_expr[[1]])
# Classes ‘tbl_df’, ‘tbl’ and 'data.frame': 330 obs. of  18 variables:
#  $ patientId         : chr  "2212" "2374" "2068" "2154" ...
#  $ age               : chr  "35" "44" "38" "66" ...
#  $ sex               : chr  "Male" "Female" "Male" "Female" ...
#  $ staining          : chr  NA NA NA NA ...
#  $ intensity         : chr  NA NA NA NA ...
#  $ quantity          : chr  NA NA NA NA ...
#  $ location          : chr  NA NA NA NA ...
#  $ imageUrl          : chr  "http://v18.proteinatlas.org/images/2763/6778_B_4_5.jpg" "http://v18.proteinatlas.org/images/2763/6778_B_5_5.jpg" "http://v18.proteinatlas.org/images/2763/6778_A_3_2.jpg" "http://v18.proteinatlas.org/images/2763/6778_A_1_2.jpg" ...
#  $ snomedCode1       : chr  "M-00100" "M-00100" "M-00100" "M-00100" ...
#  $ snomedCode2       : chr  "T-93000" "T-93000" "T-66000" "T-66000" ...
#  $ snomedCode3       : chr  NA NA NA NA ...
#  $ snomedCode4       : chr  NA NA NA NA ...
#  $ snomedCode5       : chr  NA NA NA NA ...
#  $ tissueDescription1: chr  "Normal tissue, NOS" "Normal tissue, NOS" "Normal tissue, NOS" "Normal tissue, NOS" ...
#  $ tissueDescription2: chr  "Adrenal gland" "Adrenal gland" "Appendix" "Appendix" ...
#  $ tissueDescription3: chr  NA NA NA NA ...
#  $ tissueDescription4: chr  NA NA NA NA ...
#  $ tissueDescription5: chr  NA NA NA NA ...

dir.create("img")

SLC2A3_norm <-
  SLC2A3_expr[[1]] %>%
  filter(tissueDescription1 == "Normal tissue, NOS") %>%
  filter(tissueDescription2 %in% c("Cerebral cortex", "Hippocampus", "Lateral ventricle wall"))

for (i in 1:nrow(SLC2A3_norm)) {
    download.file(SLC2A3_norm$imageUrl[i],
                  destfile = paste0("img/", SLC2A3_ab$id[1], "_",
                                    SLC2A3_norm$patientId[i], "_", 
                                    SLC2A3_norm$tissueDescription2[i], "_", 
                                    SLC2A3_norm$staining[i],
                                    ".jpg"),
                  mode = "wb")
}

SLC2A3_glioma <-
  SLC2A3_expr[[1]] %>%
  filter(tissueDescription1 %in% c("Glioma, malignant, High grade", "Glioma, malignant, Low grade", "Glioma, malignant, NOS"))

for (i in 1:nrow(SLC2A3_glioma)) {
    download.file(SLC2A3_glioma$imageUrl[i],
                  destfile = paste0("img/", SLC2A3_ab$id[1], "_",
                                    SLC2A3_glioma$patientId[i], "_", 
                                    SLC2A3_glioma$tissueDescription1[i], "_", 
                                    SLC2A3_glioma$staining[i],
                                    ".jpg"),
                  mode = "wb")
}
```

```
sessionInfo()
#> R version 3.6.0 (2019-04-26)
#> Platform: x86_64-w64-mingw32/x64 (64-bit)
#> Running under: Windows 10 x64 (build 17134)
#> 
#> Matrix products: default
#> 
#> locale:
#> [1] LC_COLLATE=English_United States.1252 
#> [2] LC_CTYPE=English_United States.1252   
#> [3] LC_MONETARY=English_United States.1252
#> [4] LC_NUMERIC=C                          
#> [5] LC_TIME=English_United States.1252    
#> 
#> attached base packages:
#> [1] stats     graphics  grDevices utils     datasets  methods   base     
#> 
#> other attached packages:
#> [1] ggplot2_3.2.0    dplyr_0.8.1      HPAanalyze_1.3.2 BiocStyle_2.12.0
#> 
#> loaded via a namespace (and not attached):
#>  [1] zip_2.0.2          Rcpp_1.0.1         pillar_1.4.1      
#>  [4] compiler_3.6.0     BiocManager_1.30.4 tools_3.6.0       
#>  [7] digest_0.6.19      evaluate_0.14      tibble_2.1.3      
#> [10] gtable_0.3.0       pkgconfig_2.0.2    rlang_0.3.4       
#> [13] openxlsx_4.1.0.1   yaml_2.2.0         xfun_0.7          
#> [16] withr_2.1.2        stringr_1.4.0      knitr_1.23        
#> [19] xml2_1.2.0         hms_0.4.2          grid_3.6.0        
#> [22] tidyselect_0.2.5   glue_1.3.1         R6_2.4.0          
#> [25] rmarkdown_1.13     tidyr_0.8.3        purrr_0.3.2       
#> [28] readr_1.3.1        magrittr_1.5       hpar_1.24.0       
#> [31] scales_1.0.0       htmltools_0.3.6    assertthat_0.2.1  
#> [34] colorspace_1.4-1   stringi_1.4.3      lazyeval_0.2.2    
#> [37] munsell_0.5.0      crayon_1.3.4
```
